# Impact of home visits to pregnant women and their spouses on gender norms and dynamics in Bauchi State, Nigeria: Narratives from visited men and women

**DOI:** 10.1177/1757975920986703

**Published:** 2021-02-01

**Authors:** Hadiza Mudi, Umar Dutse, Loubna Belaid, Umaira Ansari, Khalid Omer, Yagana Gidado, Muhd Chadi Baba, Amina Mahdi, Neil Andersson, Anne Cockcroft

**Affiliations:** 1 Bauchi State College of Nursing and Midwifery, Bauchi, Nigeria; 2 Federation of Muslim Women Association of Nigeria (FOMWAN), Bauchi State, Nigeria; 3 CIET/PRAM, Department of Family Medicine, McGill University, Montreal, Quebec, Canada; 4 Centro de Investigación de Enfermedades Tropicales (CIET), Universidad Autónoma de Guerrero, Acapulco, Mexico; 5 Bauchi State Ministry of Health, Bauchi, Nigeria

**Keywords:** maternal and newborn child health, gender transformative interventions, male involvement, gender dynamics, gender roles, decision-making, narratives, thematic analysis

## Abstract

**Background:**

Maternal and newborn child health are priority concerns in Bauchi State, northern Nigeria. Increased male involvement in reproductive health is recommended by the World Health Organization. A trial of a program of universal home visits to pregnant women and their spouses, with an intention to increase male involvement in pregnancy and childbirth, showed improvements in actionable risk factors and in maternal morbidity. We used a narrative technique to explore experiences of the visits and their effect on gender roles and dynamics within the households.

**Methods:**

Trained fieldworkers collected narratives of change from 23 visited women and 21 visited men. After translation of the stories into English, we conducted an inductive thematic analysis to examine the impact of the visits on gender norms and dynamics.

**Results:**

The analysis indicated that the visits improved men’s support for antenatal care, immunization, and seeking help for danger signs, increased spousal communication, and led to changes in perceptions about gender violence and promoted non-violent gender relationships. However, although some stories described increased spousal communication, they did not mention that this translated into shared decision-making or increased autonomy for women. Many of the men’s stories described a continuing paternalistic, male-dominant position in decision-making.

**Conclusions:**

Few studies have examined the gender-transformative potential of interventions to promote male involvement in reproductive health; our analysis provides some initial insights into this.

## Introduction

Women in Nigeria face high risks of illness and death related to childbearing, and young children in the country have high rates of morbidity and mortality (1,2). The situation is worse in Bauchi State, in the north of the country (2,3). In this region, family sizes are large, and polygamy is common (2). The main religion is Islam, with prominent Hausa and Fulani ethnicities. Women have low autonomy and low power over decisions for their health and the health of their children (4). The level of intimate partner violence is high (2,5). The government of Bauchi State identified maternal and early child health as priority concerns; the research group authoring this paper and a local non-governmental organization (NGO) undertook with them several household surveys and used the findings to support planning of services for women and children (6,7).

From 2015 to 2019, the same research group and local NGO implemented a cluster randomized controlled trial of universal home visits to improve maternal and early childhood health outcomes in the Toro local government authority (LGA) of Bauchi State. The published protocol gives details of the home visits program and the trial design (8). Increasing male involvement was an important element of the program. Female and male home visitors visited pregnant women and their spouses during pregnancy and after delivery, sharing and discussing with them the same local evidence about risk factors for maternal health, actionable at the household level: heavy work during pregnancy, domestic violence, lack of spousal communication about pregnancy and childbirth, and lack of knowledge of danger signs in pregnancy and childbirth (9). In repeated visits, the home visitors shared evidence about the harmful effects of the identified risk factors and separately discussed with pregnant women and their spouses what they could do, and were doing, about these risks. They shared information about danger signs in pregnancy and the need to seek care from health facilities, and about the benefits of breastfeeding and childhood immunization. Quantitative analysis of the trial found that women in the visited areas had significantly fewer complications in pregnancy and after birth than women in non-visited areas, as well as improvements in the four identified risk factors (10); child outcomes also improved in the visited areas (11).

The home visits program had the potential to be gender-transformative because it addressed maternal health risk factors strongly related to gender norms and dynamics. Reducing heavy work during pregnancy questions the traditional division of labor, increasing spousal communication impacts shared decision-making, and protection from gender violence is a fundamental aspect of women’s dignity and rights. The home visitors invited pregnant women and their spouses to think about their behaviors and about social and cultural norms which could be harmful. In addition to the quantitative measurement of the impact of the home visits, we collected narratives of change from women and men in visited households, to understand what the visits meant to them. We describe here an analysis of the stories to specifically explore the impact of the home visits on gender roles and decision-making and to evaluate to what extent the intervention was gender-transformative.

## Methods

We used purposive sampling to identify female and male story tellers. Home visitors and their supervisors proposed women and men in households who had received home visits and who could be approached to tell their stories. Almost all the women they proposed had already given birth after being visited several times during pregnancy, and the men they proposed had been visited several times during the pregnancy of their spouses. Drawing on the most significant change technique (12), four trained fieldworkers (two women and two men) collected stories of change from 23 women who had been visited during their pregnancies and 21 men who had been visited during their wives’ pregnancies. The fieldworkers were all from Bauchi and spoke the Hausa language. After seeking oral informed consent and ensuring privacy for the interaction, they asked the respondents: ‘Please tell me a story describing what you think is the most significant change in your life since we started visiting you in the home visits program. Your story could be about a positive change in your life, or about a negative change’. They took notes while the story tellers spoke and then read back the stories to the story tellers to check for accuracy. They then translated the recorded stories into English. HM, UD (local female and male trained qualitative researchers), LB (female Muslim anthropologist) and AC (female senior researcher) conducted an inductive thematic analysis of the stories. Applying a gender lens, each of them identified themes and organized them into a coding structure. They discussed and agreed upon a common coding structure and applied it to the stories. The gender lens refers to applying a gender analysis (13). In this case, we focused on changes in gender roles and decision-making attributed to the home visits.

### Trustworthiness

Our strategies to enhance trustworthiness (14) were based on criteria proposed by Guba (15). We ensured credibility by using validated research methods to collect and analyze data, by triangulation between stories from women and men, by the qualifications and experience of the researchers, and by member checking with the story tellers and the local research team. We promoted transferability by providing a description of the participants and context of the study. Our description of the research processes supports dependability (corresponding to the concept of reliability in quantitative research). We increased confirmability through reflexivity; during debriefing sessions, the research team members discussed their own biases, assumptions, beliefs, and suppositions that might affect their interpretation of findings.

## Results

### Characteristics of the participants

All the story tellers were married, with a mean age of 32 years among the women and 40 years among the men. Most were in polygamous families. The women had from 3 to 8 children and the men 1 to 14 children. None of the women had more than a primary education; the men had primary or secondary education. A few women were engaged in petty trade and men reported occupations in public services, petty trade and farming.

### Heavy work during pregnancy

Men and women gave different responses to the information about the harmful effects of continued heavy work during pregnancy shared in the home visits. Some women described how the home visits led them to decide for themselves to stop heavy work during pregnancy:

As a result of my discussions with your health worker, I decided to reduce my domestic activities and to concentrate on nursing my baby in my womb. (25-year-old woman)

From the men’s perspective, the visits led them to stop assigning their wives to heavy work during pregnancy:

Before I used to force my wife to wash all my clothes and engage in heavy exercise during her pregnancy in the name of homework. That resulted in her experiencing difficulties during childbirth. I now understand that it [heavy work] is bad and it creates serious complications during pregnancy and childbirth. This knowledge led me to stop her from engaging in any form of heavy work. (30-year-old man)

None of the women’s or men’s stories explicitly described a change in division of labor or men being more engaged in heavy household tasks normally undertaken by women.

### Use of health services

Men’s narratives suggested that the information shared with them by the male home visitors made them more knowledgeable about pregnancy complications and more supportive of their wives using health services during pregnancy. Their stories described how they now instructed or encouraged their wives to visit services, indicating that they retained the role of decision-maker in these matters:

Previously, I would stop my wives from attending antenatal care services. Now, I have started encouraging my wives to visit antenatal care offered by government health facilities. (39-year-old man)

Just this morning she told me she was not feeling well. I told her that she must visit the health facility. I also advised her to check the pamphlet and find out if she was suffering from any danger sign mentioned there. (30-year-old man)

Some women’s narratives suggested they had autonomy to decide to use health services. It was not clear if this autonomy was new or pre-existing:

I decided to take my baby for immunization. My baby is now seven months and has received five immunizations. (28-year-old woman)

Other women described how the husband remained the decision-maker about the use of health services, one explaining that her husband now *allows* immunization of their children:

He [husband] forbade me from taking our children to the health facility for immunization. I had to obey his order because he is the head of my family. Things changed for us last year … A male worker engaged my husband in discussions and gave him awareness. *Alhamdulillah*! My husband accepted his advice. Now he allows our children to be immunized. (40-year-old woman)

### Spousal communication

The increased discussion about pregnancy and related matters among visited couples reported by the previous quantitative analysis of the impact of the home visits (10) was reflected in the narratives of change, especially narratives from men. They described both a greater ability on their side to talk to their wives, and a greater ability of their wives to approach them and share ideas about pregnancy and other matters. Some of the language in the stories suggested that this increased discussion between husband and wife sometimes led to shared decision-making:

All through my life, I never gave any time to my wife. Many times, she would not even come close to me because she would be afraid of me and worried about how I would react… I was forced into thinking that I had to change my attitude. I started listening to my wife. I would hear from her on many matters concerning our family and even other issues that needed to be discussed. (58-year-old man)

Previously, I was too shy to sit down with my husband and discuss my pregnancy with him. I can now sit down freely and discuss with my husband about our family issues. (30-year-old woman)

### Gender violence

Some narratives of men suggested that their discussions with male home visitors about the effects of gender violence on maternal health led them to reflect on gendered norms, and to change their views and behavior:

I believed that domestic violence was a normal occurrence between a husband and his wife. When the home worker started visiting me, I was able to understand that domestic violence is not good for a pregnant woman. I learned how to live with my family in peace and harmony without any domestic violence… I feel satisfied, normal, and respected. (60-year-old man)

## Discussion

Our analysis of the stories of change from visited women and men suggests that the visits succeeded in engaging men to improve maternal and child health: men felt more knowledgeable about the risks and took actions to reduce them, for example by stopping their pregnant wives from undertaking heavy work, or by allowing their children to be immunized. The home visits program increased men’s support for antenatal care, immunization, and seeking help for danger signs, it increased spousal communication, and it led to changes in perceptions about gender violence and promoted non-violent gender relationships (16).

The impact of the home visits on decision-making and women’s autonomy was not clear cut. The increased spousal communication described in some stories did not necessarily translate into shared decision-making or increased autonomy for women. Some women’s stories reflected autonomy to adopt safer behaviors (such as reducing heavy work in pregnancy), but it was not clear that this autonomy was a result of the home visits. In Bauchi, women are generally not allowed to leave home without the permission of their husband or guardian. This limits their autonomous access to health services, even if they have their own financial resources to travel and to pay for care. Women’s participation in decision-making about their health care and other matters is much lower in Bauchi than the national average in Nigeria (2). Many of the men’s stories described a continuing paternalistic, dominant position in decision-making. This is reflected in the language used: ‘I allow my wife to…’, ‘I told her she must visit the health facility’.

Previous studies of increasing male involvement in reproductive health have reported mixed effects on shared decision-making and women’s autonomy. A study in India to increase male involvement reported increased discussion between spouses after delivery and increased shared decision-making about reproductive health and health care seeking (17). A similar study in South Africa reported increases in spousal discussion about sexual relations, immunization, and breastfeeding, but not in male partner supportive behavior (18). In rural India, pregnant women whose husbands attended group meetings reported increased help from them with household tasks, and women receiving home visits reported an increase in family discussions about birth-preparedness (19). A workplace intervention in Turkey to increase male involvement in reproductive health reported increased men’s participation in discussions with their partners; but some women reported that their partners used their increased knowledge to dominate decision-making (20).

The stories in our study provided insights into how the home visits impacted gendered division of labor. Some women described deciding for themselves to reduce heavy work in pregnancy, and men described ensuring that their wives did not undertake heavy work in pregnancy. Perhaps some men took over some of the household work from their wives, although no stories specifically described this. Neither women nor men suggested they had changed their views about what is ‘women’s work’. Such views are likely to be deeply ingrained and not questioned by either women or men. The approach in the home visits was not to prescribe actions; the households themselves chose what changes they made in the light of the local evidence shared by the home visitors.

The World Health Organization recommends interventions to increase male involvement for promotion of maternal and newborn health but cautions that such interventions should be implemented in a way that ‘respects, promotes and facilitates women’s choices and their autonomy in decision-making’ (21). A 2014 review of gender-integrated interventions for maternal and child health reported that most had mixed effects on gender dynamics and health outcomes (22). A secondary qualitative analysis of studies in a systematic review of male involvement for maternal and child health noted a lack of studies examining the gender-transformative potential of male involvement in interventions (23,24). Most of the included studies were from South Asia. Our analysis of the stories of men and women reached by the home visits program in Bauchi State is a modest contribution towards closing this evidence gap, particularly in West Africa.

### Limitations

The women and men who gave their stories were not a representative sample of the visited women and men; for the purposive sample, the home visitors identified visited women and men who ‘had a story to tell’. The selection of story tellers is a recognized issue when applying the most significant change technique (25). The stories do not indicate the usual or average effects of the home visits; rather they illustrate what changes the visits can produce for some people. The fieldworkers took notes rather than audio-recording the stories. Some people consider audio-recording improves data reliability, but our experience in resource-poor settings accords with that of other authors: people speak more freely when they are not being audio-recorded (26). We did not collect the stories specifically to assess the impact of the home visits on gender norms and dynamics. They were intended to provide insights into how pregnant women and their spouses perceived the home visits, and to allow us to examine the mechanism of the impact of the visits, in terms of the theory of change for the intervention, which included intermediate outcomes between knowledge and action (8). We could not examine the full range of gender-related outcomes, but the stories offered some useful pointers about how the visits affected shared decision-making, women’s autonomy, and gender roles.

## Conclusion

The stories of change from women visited during their pregnancies and from the husbands of visited women suggest that the visits increased male involvement and led both men and women to take actions to improve maternal and child health. However, the stories did not indicate that the visits increased women’s autonomy or their role in decision-making.
